# Illusory Streaks from Corners and Their Perceptual Integration

**DOI:** 10.3389/fpsyg.2016.00959

**Published:** 2016-06-23

**Authors:** Sergio Roncato, Stefano Guidi, Oronzo Parlangeli, Luca Battaglini

**Affiliations:** ^1^Department of Psychology, University of PadovaPadova, Italy; ^2^Department of Political, Social and Cognitive Sciences, University of SienaSiena, Italy

**Keywords:** illusions, optical illusions, phantom lines, grouping factors, contour perception, hermann grid, filling-in

## Abstract

Perceptual grouping appears both as organized forms of real figural units and as illusory or “phantom” figures. The phenomenon is visible in the Hermann grid and in configurations which generate color spreading, e.g., “neon effects.” These configurations, generally regular repetitive patterns, appear to be crossed by illusory bands filled with a brighter shade or a colored tinge connecting the various loci of illusory effects. In this work, we explore a particular new illusion showing a grouping effect. It manifests as illusory streaks irradiating from the vertexes of angular contours and connecting pairs of figures nearby. It is only clearly visible when more than one figure is shown, and takes the shape of a net crossing their corners. Although the grouping effect is vivid, the local source of the illusion is completely hidden. Theories explaining this effect as due to the irradiation of illusory streaks (mainly that of Grossberg and Mingolla, 1985a,b) do not fully explain the figural patterns presented here. Illusory effects have already been documented at the angles of various figures, causing them to alter in amplitude and brightness; however, the figure illustrated here appears to have different features and location. Phenomenological observations and an experiment were conducted to assess the role played by geometric and photometric parameters in this illusion. Results showed that sharp angles, in low contrast with the surround, are the main source of the illusion which, however, only becomes visible when at least two figures are close together. These findings are discussed with respect to theories of contour processing and perceptual grouping, and in relation to other illusions.

## Introduction

Since the analyses of the early Gestaltists (e.g., Wertheimer, 1923), perceptual groups and objects are known to be the results of integration/interpolation processes occurring at different stages: stimuli in the visual field are either summarized or mediated, producing the perception of higher-order figural units.

Vision research on figural grouping, and more specifically approaches based on phenomenological accounts of visual perception, have revealed the role played by several factors in perceptual grouping, such as the well-known gestalt rules of orientation, closure, and symmetry. However, the role of photometric variables such as stimulus luminance, processed during earlier stages of the visual system, are still difficult to understand, probably due to our still incomplete understanding both of what light is and of the psychophysiology of early vision processes.

Study of early processes in perceptual grouping has mainly focused on the role of contrast polarity, with a wide variety of tasks and stimuli: contour formation in Glass patterns (Glass and Switkes, 1976; Burr and Ross, 2006), illusory contour formation (Prazdny, 1983; Dresp and Grossberg, 1997; Spehar, 2000), line contrast detection (Wehrhahn and Dresp, 1998), orientation discrimination with co-linear lines (Brincat and Westheimer, 2000), perceptual closure (Spehar, 2002), contour detection (Field et al., 2000), illusory misalignment and tilt of contours (Kitaoka et al., 2004; van Lier and Csathó, 2006; Guidi et al., 2011), and contours reassembling in perceptual transparency (Kitaoka et al., 2001; Singh and Huang, 2003). The results of these studies reveal considerable differences in sensitivity for contrast polarity in the figural contexts examined, so that we are far from having a proper view on the extent to which this factor contributes. Each stimulus configuration appears to produce results which are difficult to generalize to other configurations. Therefore, despite the number of studies, still little is known about the influence of luminance contrast on perceptual grouping.

**FIGURE 1:**
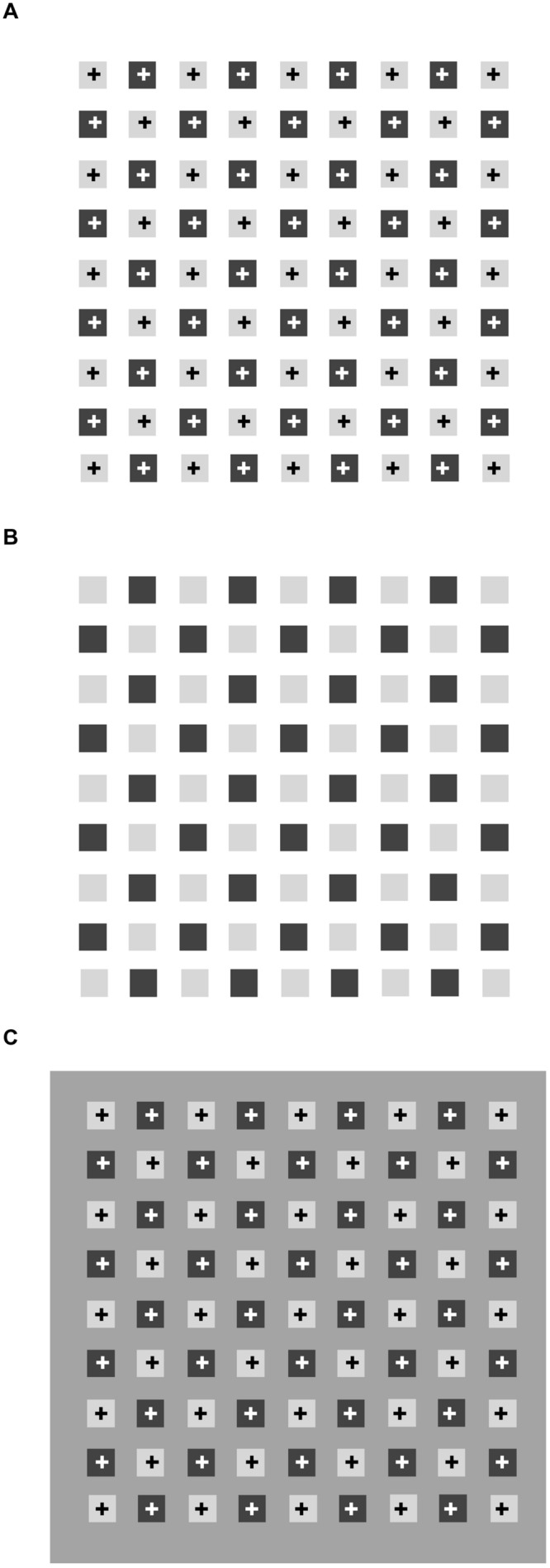
**Same pattern of small squares reproduced three times. (A)** With “+” inside each square, **(B)** with “+” erased, **(C)** against a darker background. Diagonals in **(A)** are illusory effects, also visible in **(B)**, but less vividly.

The role of luminance contrast may be better understood by distinguishing two kinds of phenomenally important grouping processes: (1) a new perceptual unit results from the combination of differing elements (e.g., a square made of four different segments); (2) the grouping of elements is mediated by the generation of additional elements connecting various figures, such as a lattice made of neon light spreading through the elements and connecting them (Varin, 1971; Van Tuijl, 1975; Grossberg, 1984). **Figure 1A** shows these illusory effects as dark and pale gray squares, separated by regular intervals, arranged in a checkerboard-like pattern. Dark diagonal streaks appear to cross the brighter squares along the directions of the bisecting angles. These streaks are illusory and when, as in **Figure 1B**, the inner crosses are removed, the illusion is weakened. Instead, with a darker background (**Figure 1C**), the phantom streaks can no longer be seen.

In explaining both these grouping effects, Grossberg and Mingolla (1985b) exploited Gestalt psychology on the separation between figures and the background, hypothesizing three interconnected systems. The first, the Boundary Contour System (BC system), pre-attentively generates boundary representations starting from differences in oriented luminance and color edges in the stimuli. The second, the Feature Contour System (FC system), operates in parallel with the BC System, and is presumed to be responsible for representing the visible features of surfaces (e.g., color, brightness, etc.). It not only receives the same bottom–up signals which feed the BC System (stemming from detection, in the earlier stages of visual processing, of oriented luminance or color discontinuities) but also recurrent feedback from the BC System, in the form of organized boundary representations coding figure-background status. Grossberg and Mingolla (1985b, p. 143) state: “The FC signals here initiate the filling-in processes whereby brightness and colors spread until they either hit their first boundary contour or are attenuated by their spatial spread.” Lastly, a third system, the Object Recognition System (OR SystemS), exchanges learned information with the BC System, so that both can change what was pre-attentively processed and affect the activity of the FC System.

**FIGURE 2:**
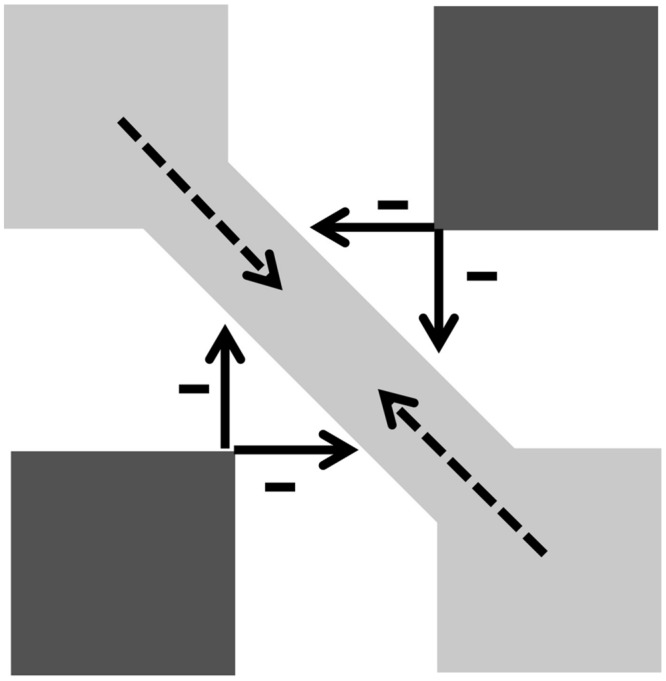
**From model of Grossberg and Mingolla (1985a,b).** Less contrasted gray-to-white edges “activate orientation receptive fields less than does each black square.” Arrows: inhibition of strongly activated vertical and horizontal tuned cells on nearby weakly activated cells. Gray-to-white contours: inhibition in both orthogonal directions: “This conjoint vertical and horizontal inhibition generates a gap within the boundary contours at each corner of every light-gray square” (p. 176). Second-stage disinhibition gives rise to diagonal boundary contours, those originating from opposite corners conjoin to form stronger contours (modified from Grossberg and Mingolla, 1985a,b). Reprinted with permission of authors and publisher.

To explain a phenomenon like that shown in **Figure 1B**, a pattern originally created by Beck et al. (1983), in which pale gray diagonal bands are seen joining the angles of pale gray squares, Grossberg and Mingolla (1985a,b, p. 156) examined two properties of the BC System: “[…] the contrast-sensitivity of the oriented receptive fields and the lateral inhibition within the first competitive stage among like-oriented cells at nearby positions.” In their view, during this stage, the highly contrasted oriented luminance discontinuities at the borders of the dark squares, strongly activating responsive luminance-edge cells in the BC System, trigger lateral inhibition signals to nearby cells tuned to the same orientation and contrast polarity. Activation in these cells, already weak due to the low contrast of the edges of the pale gray squares, is thus turned off, preventing boundary closure in the corners of the squares (**Figure 2**). By cooperation between contour generation and lateral inhibition processes, diagonal boundary contours are generated at a second stage of figural segregation, once boundary assignment is determined, and can contain the spreading of signals carrying featural information in the FC System.

“The lattice of diagonal boundary contours enables gray featural quality to flow out of the squares and fill in the positions bounded by the lattice within the FC System” (Grossberg and Mingolla, 1985b, p. 156).

**Figure 1A** shows illusory streaks connecting the pale gray squares having higher luminance and lower contrast with the white background. Grossberg and Mingolla’s account of the pattern of Beck et al. (1983) in Figure 1B can be extended to Figure 1A, in that it is an exact replica with small outline symbols inside. In both patterns, the same figural and photometric conditions are met for the illusory diagonals to appear.

**FIGURE 3:**
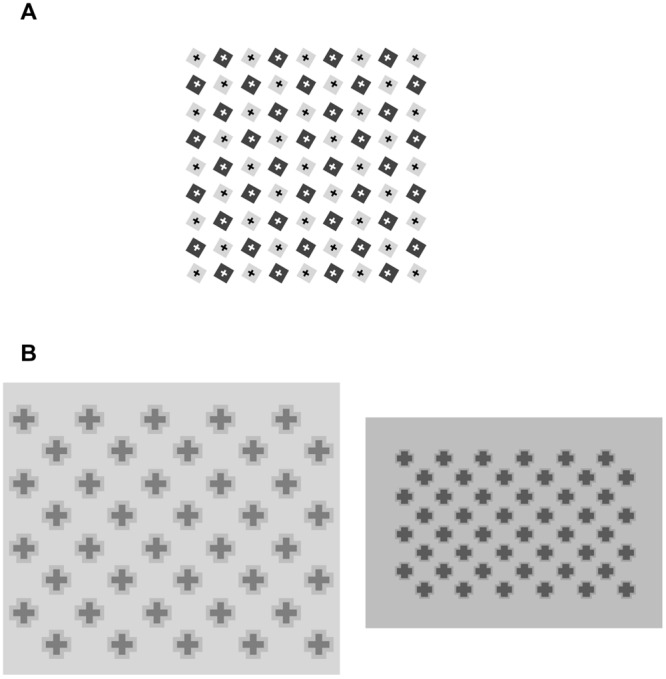
**(A)** Same pattern as in **Figure 1A**, with squares rotated 30°; **(B)** Identical cross-like figures arranged in orthogonal rows and columns. Streaks diagonally connecting figures are illusory effects.

Some simple manipulations of these patterns show that the alternation of light and dark components is not a necessary condition for the effect to arise. In **Figure 3A** the squares are rotated by 30°, which leaves the overall organization intact: we therefore expect to see the same illusory streaks arising in **Figure 1A** connecting the diagonal arrays of light gray squares. Actually, we see a homogeneous pale gray filling the interspaces, and could again conclude that alternating light and dark linear arrays is a sufficient condition to produce phantom streaks. However, it is not a necessary condition, as the following illustration shows. **Figure 3B** shows a cross-like figure replicated several times to form a regular array of diagonally aligned shapes. Here, most observers say they see thin illusory bands in the interspaces connecting the corners of each cross with the corners of the surrounding ones. The figures have identical shapes and colors, so that the luminance contrast does not vary across the borders. In this configuration, according to Grossberg and Mingolla (1985b), the isochromatic figural units should not generate illusory contours, but this prediction is not confirmed by the appearance of a lattice of gray streaks joining the corners of the figures.

**FIGURE 4:**
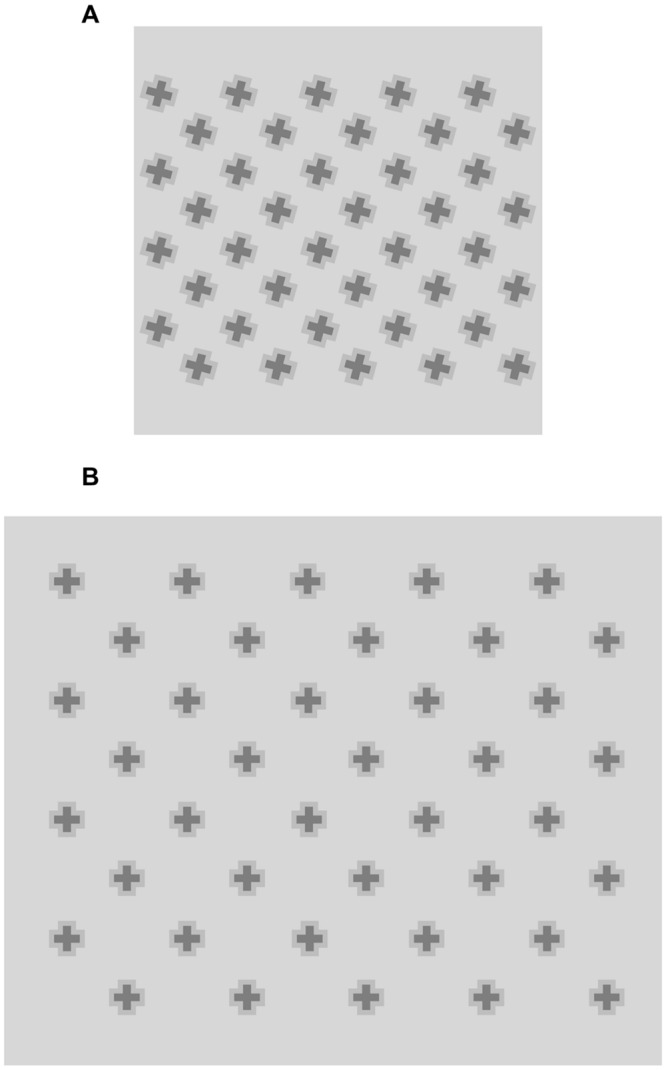
**(A)** Same as **Figure 3B** with crosses rotated 15°. **(B) Figure 3B** with spaced crosses.

This new configuration is suitable for testing further manipulations. **Figure 4A** shows the crosses rotated by 15°, which weakens the illusory effects or makes them disappear entirely. The same detrimental effect is produced by increasing the distance between the crosses, as in **Figure 4B**, where again we do not see phantom bands passing through the wider spaces between the figures.

**FIGURE 5:**
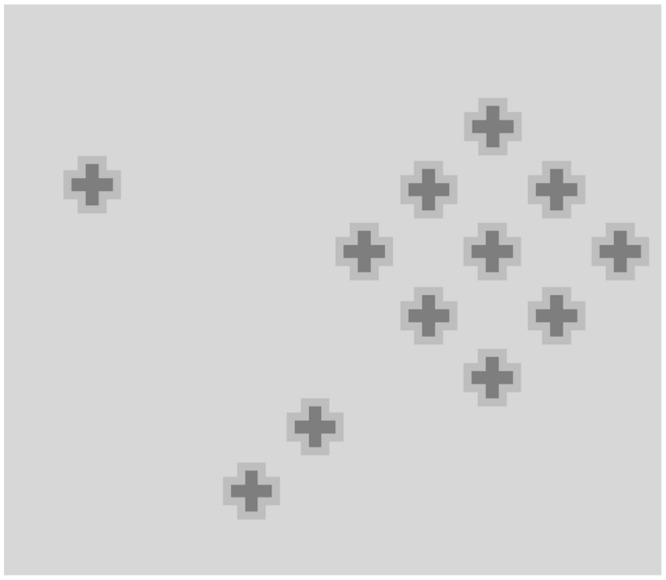
**A single cross, two crosses, and group of crosses.** Illusory effect is only clearly visible between grouped crosses.

Grouping of the inducing figures seems to be necessary for illusory lines to emerge. **Figure 5** in fact shows that no illusory effect stems from the same crosses of **Figure 3B** when they are displayed in isolation. With pairs of crosses, the effect is still difficult to perceive, but it becomes clearly visible when various crosses are phenomenally aggregated in a group.

To conclude, illusory lines appear to join the vertexes of angular perimeters, provided that these corners are *aligned* and sufficiently *close*. This leads us to hypothesize that the illusory diagonals originate at the corners of the figures as short phantom contours oriented so that they bisect them. Luminance patterns at the corners trigger changes in brightness, taking the form of oriented lines or streaks connecting the vertexes. They are not visible when the sources are in isolation but, once they are sufficiently close in distance and orientation, they emerge as a group, as Gabor shapes aligned along a contour become detectable and “pop out” within a field of identical, randomly-oriented, Gabor shapes (Field et al., 1993).

**FIGURE 6:**
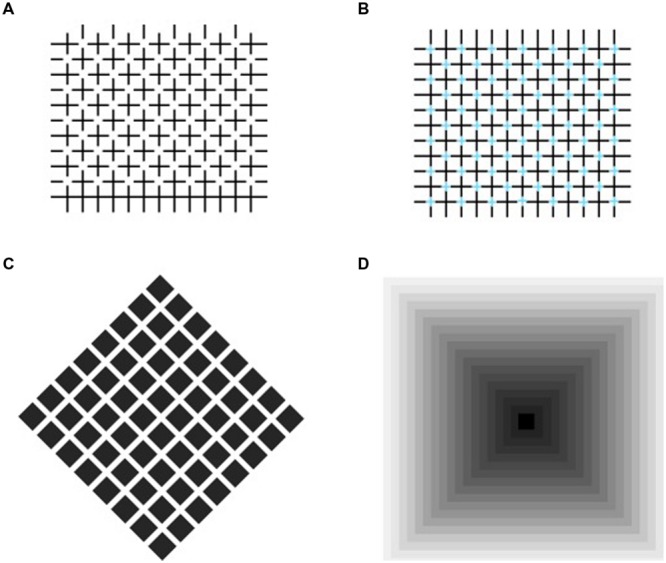
**Illusions with elements with different brightness levels. (A)** Diagonal bands appear to connect illusory disks where perpendicular lines cross; **(B)** Intersections of lines are replaced by colored crosses, producing neon light diffusion; **(C)** Brightness alterations observed in interspaces of Hermann grid (Prandtl, 1927); **(D)** Angular surfaces (Vasarely’s Arcturus).

The grouping effect related to perceptual illusions involving elements of various brightness levels is well-known (see **Figure 6**). The illusory diagonals in **Figures 1A** and **3B** may have the same origin as the illusory lines or “streets” in the patterns of Ehrenstein (Redies and Spillmann, 1981; Zucker and Cavanagh, 1985; Kitaoka, 2001; Hamburger et al., 2012) and those generated as neon lights. In the former (**Figure 6A**), perpendicular black lines are erased in the crossing regions, eliciting the perception of disks of a brighter shade than the surround, and diagonal bands connecting the illusory disks in a “street” of equally enhanced brightness. When the intersections of the lines, instead of being erased, are replaced by colored crosses (**Figure 6B**), a vivid impression is elicited and colored bands appear to pass through the regions of neon diffusion (Van Tuijl, 1975). Similarly, in **Figure 1A**, a local unknown illusory spreading of color may group with collinear identical effects and give rise to phantom diagonals.

The phenomenon shown in **Figures 1A** and **3B** probably originates in the corner vertexes, but phenomenological observations do not provide convincing causes. What we perceive here may be described as color spreading from the vertexes, but why an angle becomes an open gate to allow color inside the square to flow out and fill the background does not seem to match the theory of Grossberg and Mingolla (1985b). In addition, alterations in brightness have been observed both on angular surfaces (Vasarely, 1970; Schachar, 1976; Morgan, 1996; Troncoso et al., 2005, 2009) and in the interspaces of the Hermann grid (Hermann, 1870; Prandtl, 1927), but neither their loci (**Figures 6C,D**) nor the photometric conditions coincide with those in which the illusions in **Figures 1A** and **3B** arise.

The phenomenon of perceptual grouping shown in **Figures 1A** and **3B** thus appears as a new illusion which cannot be explained by present theories. To determine more precisely the conditions giving rise to this phenomenon, we conducted an experiment to test the effects of systematic variations of corner amplitude and luminance contrast at their sides.

**FIGURE 7:**
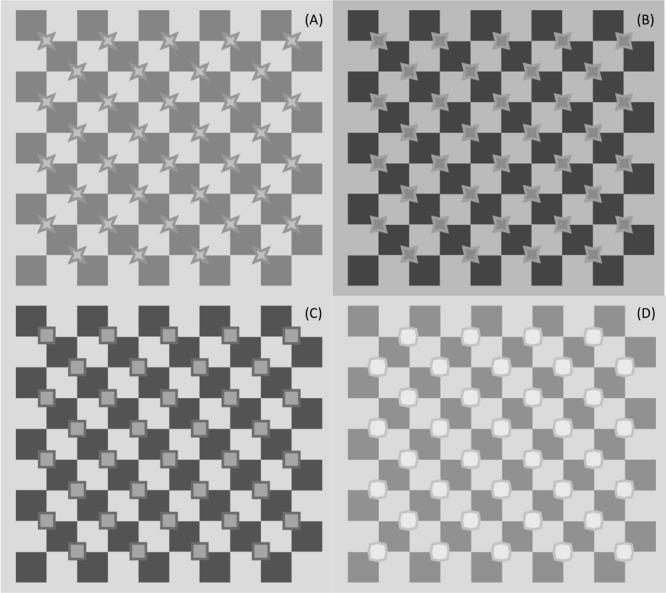
**Four types of experimental stimuli, one for each angle amplitude at corners. (A)** acute angle (46°), **(B)** acute angle (62°), **(C)** square angle (90°), **(D)** obtuse angles (alternating 110° and 160°).

**FIGURE 8:**
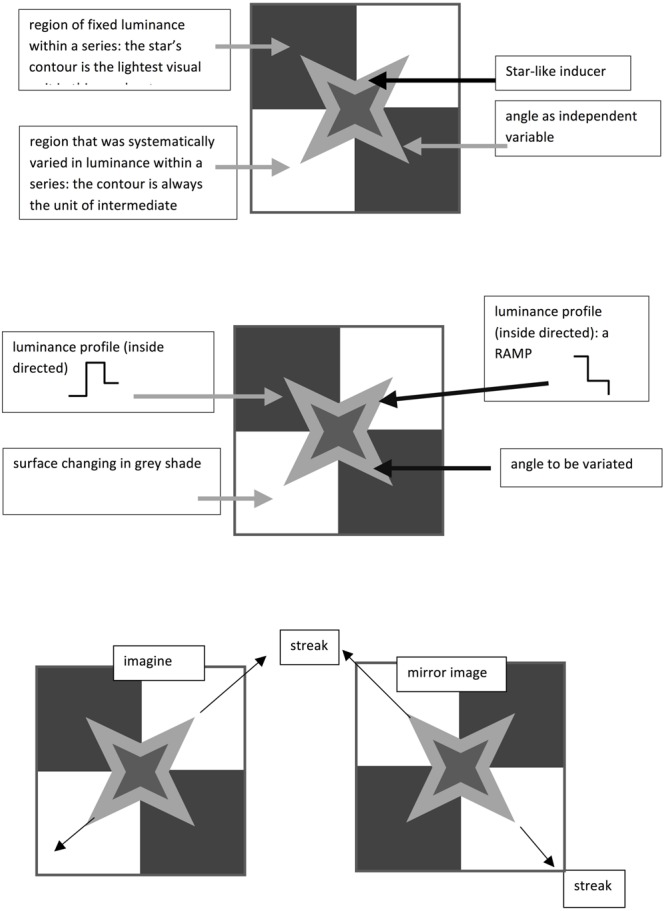
**Close-up of crossing regions in **Figure 7**.** Local patterns enlarged to illustrate independent and dependent variables.

The experimental stimuli were variations of a general pattern, in which a series of filled polygons were placed over the X-junctions of a checkerboard (see **Figure 7**). These polygons (henceforth *inducers*), although they have quite different shapes, are actually derived from a common basic pattern – an octagon in which angles of two amplitudes alternate along the contour. For example, alternating 60 and 210° angles form a four-tipped star-like figure, alternating 90 and 180° angles form a square, and alternating 120 and 150° angles produce an octagon. This means that differing angle widths can be tested. In addition, polygon contours create differing luminance profiles on the checkerboard (**Figure 8**), allowing us to test the luminance conditions in which a corner acts as a source of color spreading.

The illusory effects perceived in **Figure 7** are the first demonstration that the figural configuration we created is a good tool to study the geometric and photometric influence on the illusion. We can see streaks of different polarity (**Figures 7A,B**) and match them with the shade of the inner surface: streaks seem to be irradiating from lighter inducer surfaces and vice versa. They do not seem to be present in the quadrants, where the contours have extreme luminance (being either the lightest or darkest regions in the quadrant), but they do connect corners having a *luminance gradient* at the border. This pattern thus turned out to be suitable to document both the effects of corner amplitude and luminance contrast. The particular location of the octagons, at the center of four quadrants of opposite luminance, allows testing of the effects of different luminance profiles at borders.

The experiment reported in the following section was conducted to test the effect of these two variables systematically. We concentrated on the angular regions of the contour of the figure, to demonstrate that systematic variations of the geometric variable (angle amplitude) and photometric factors (contrasts at borders) affect the visibility of illusory streaks.

## Experiment

The experiment aimed at measuring the vividness of illusory streaks and how it is affected by geometric and photometric variables. The stimuli used were the same as those shown in **Figure 7**: checkerboards with inducers of various shapes overlying the X-junctions. **Figure 8** shows some of these local patterns, enlarged to highlight independent and dependent variables.

The effects of the following parameters were tested:

(a) Corner amplitude. According to previous research results, we expect stronger illusory effects with sharper angles.
(b) Contrast magnitude. In **Figure 1A**, the illusory streaks were seen to fan out from low-contrast corners: systematic observations may confirm whether these findings can be generalized.
(c) Contrast polarity. The streaks were expected to have the same contrast polarity as the corner surface: we should perceive a beam of light propagating from an angular surface lighter than the surround.
(d) Luminance profile. A luminance gradient at the external profile of the star-like figure was predicted to strengthen the illusion; the opposite, i.e., a contour of extreme luminance, is expected to prevent surface color spreading.

### Methods

#### Subjects

The participants in the experiment were one of the authors and 19 psychology students of the University of Siena (12 women, aged 20–25 years) who were unaware of the aim of the experiment. All participants had normal or corrected-to-normal visual acuity. All gave their written informed consent in accordance with the Declaration of Helsinki, and were debriefed at the end of the experiment about the purpose of the study.

#### Design and Stimuli

The stimuli were generated by manipulating two variables: the geometrical shape of the inducers, and the luminance of their borders and inner surfaces.

#### Geometrical Variables

**FIGURE 9:**
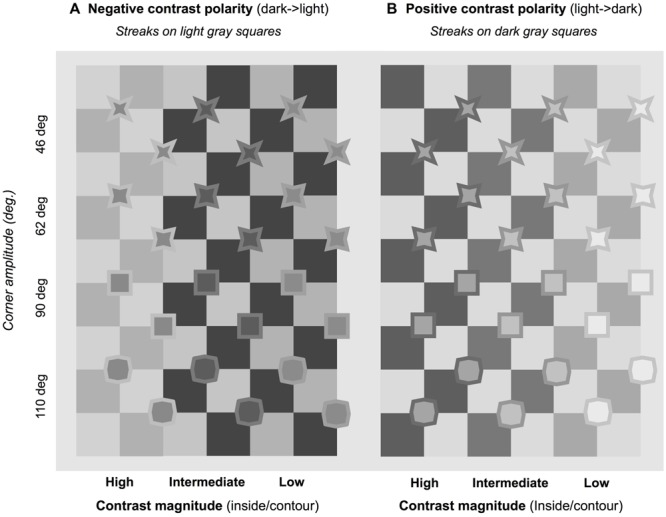
**Examples of experimental patterns.** Streaks in negative contrast polarity **(A)** oriented diagonally from top-left to bottom-right; in positive contrast polarity **(B)** oriented from bottom-left to top-right. In neither case do streaks appear visible with inducers having obtuse angles (e.g., in lower part of figure).

Four types of inducers were used:

(a) Star-like (acute): 46° of acute angle amplitude (**Figures 7A–9**);
(b) Star-like (acute): 62° acute angle amplitude (**Figures 7B–9**);
(c) Squares: 90° amplitude (**Figures 7C–9**);
(d) Octagons: alternation of 110 and 160° angles (**Figures 7D–9**).

#### Photometric Variables

The luminance magnitudes of the inducer surfaces and the contours were combined, to create an inducer-contour-background ramp both increasing and decreasing in luminance. **Table 1** lists the six pairs of surface-contour luminance values and their corresponding Michelson contrast levels. Darker surfaces were bordered by three lighter contours, and lighter surfaces were bordered by three darker contours. Examples of these patterns are shown in **Figure 9**. Luminance levels were measured on a Minolta LS-100 luminance meter. For each shade of gray used in the stimuli, nine luminance measures were taken at different positions along a grid, inside a square patch in the center of the screen.

**Table 1:** Six combinations of contour/inner surface luminance in inducers (cd/m^2^) and corresponding Michelson contrast.

Photometric condition	Inner luminance (cd/m^2^)	Contour luminance (cd/m^2^)	Michelson contrast	Contrast magnitude class	Contrast polarity
1	10,00	24,00	-0,41	High	Negative (dark→light)
2	4,40	7,60	-0,27	Intermediate	
3	10,4	14,7	-0,17	Low	
4	16,50	6,20	0,45	High	Positive (light→dark)
5	25,00	12,60	0,32	Intermediate	
6	37,50	25,20	0,19	Low	

*In photometric conditions 1–3, direction of contrast variation is from dark to light, and vice versa in conditions 4–6.*

Twenty-four inducers were generated by combining the photometric variable (six levels) with the geometrical variable (four levels). These were drawn on two checkerboards, one the “negative” reproduction of the other. The introduction of these two sets of stimuli allowed us to monitor the same illusory effect, both in one direction (clockwise inclination) and in the mirror one (anti-clockwise inclination), for a total of 48 stimuli. **Figure 9** shows the 24 inducer couples, to illustrate these conditions. The streaks in negative-contrast polarity (**Figure 9A**) run diagonally from top-left to bottom-right, and those in positive-contrast polarity (**Figure 9B**) from bottom-left to top-right.

A series of variations for each of these stimuli was generated by gradually changing the luminance of the checkerboard tiles, from lighter to darker and vice versa. The creation of each series of stimuli followed two steps. In the basic starting configuration, the dark and light tiles were very different in luminance, care being taken that no streaking line was perceived. A second stimulus was then depicted, varying the luminance of the tiles where the inducers formed a luminance ramp. The operation was replicated, with a further variation in shade, to obtain a third stimulus, and so on until the luminance gradient was replaced by a luminance step. The luminance varied in regular steps of magnitude in the descending (white-to-black) and ascending (black-to-white) ramps (from the inside of the inducer to the squares of the checkerboard, passing through the contour). In the black-to-white ramp, the variable surface increased/decreased in luminance in steps of 2.0 ± 0.2 cd/m^2^; in the white-to-black ramp, the variation was in steps of 0.6 ± 0.3 cd/m^2^. The 48 series of stimuli were reproduced in slides shown to the observers twice in fixed order: once in ascending (darkest to lightest shades) and once in descending order (Supplementary Material).

The stimuli were presented on a 17″ CRT NEC MultiSync 95F monitor with 1280 × 1024 pixel resolution (mean luminance, 26.9 cd/m^2^, horizontal refresh rate 96 Hz, vertical refresh rate 160 Hz), powered by a PC. Viewing distance was 80 cm. Overall, the stimulus subtended 12.8° of visual angle, both horizontally and vertically. The sides of the checkerboard squares were 1.8 cm long and subtended a visual angle of 1.29°. The inducers were inscribed in a circle subtending 1° of visual angle. The contour was 1.4 mm thick.

### Procedure

The method of limits was used to determine the luminance of the corner surrounds required to perceive illusory streaks. After each stimulus presentation, subjects were asked to say whether they perceived diagonal streaks and, if so, to indicate the direction of their inclination by saying “left” or “right,” and reporting polarity (lighter or darker than the background). For each of the 96 trials (each corresponding to one series), two luminance magnitudes were calculated: one in which the illusion began to appear (T1), and one coinciding with the illusion vanishing (T2). The difference between T1 and T2 corresponds to the range of luminance variation during which the illusion persisted, and was considered as a measure of its vividness. The experiment was divided into two sessions over two successive days, and subjects were told that they could stop participation at any time during the sessions without giving any reason.

At the beginning of the experiment, participants were presented with a stimulus and given the following instructions: “The figure displayed here is made up of a checkerboard and some regular figures overlapping the corners. After the gray shades change, some streaks may appear, crossing the checkerboard diagonally.” The experimenter then showed some examples until the observers convincingly reported seeing light or dark gray streaks, and then continued: “Each trial starts with the presentation of a figure. Please say whether you perceive the streaks and, if so, indicate their polarity (lighter or darker than the background) and inclination, clockwise or anti-clockwise. After this first experiment, a second one with a different checkerboard will follow.”

Some practice trials were carried out. Subjects were instructed to indicate both polarity and inclination as soon as they perceived the illusory effect and, if it persisted in the following stimuli, then only the direction. The experimenter recorded the “right” or “left” response and interrupted the trial following two consecutive reports with no diagonal perceived.

The figures were displayed on a CRT monitor and lasted for 1 s. Immediately after the presentation of each configuration, a mask stimulus composed of randomly arranged geometrical figures and lines was displayed for 1 s. Observers were asked to give their responses within this 2-s interval. The stimuli were presented by MS Powerpoint, and both responses and thresholds were manually recorded by the experimenter. Not all the programmed trials were run. Conditions in which the inducers were octagons with obtuse angles did not elicit any illusory streaking effects. The experimenter only tested six of the planned series of 24 stimuli.

### Results

#### Direction of Illusory Effect

There was total agreement among subjects’ judgments: the illusory lines were seen to cross the checkerboard diagonally, and appeared as prolongations of the corners formed by the contours of intermediate luminance (i.e., a luminance ramp).

#### Polarity of the Illusory Effect

The illusory streaks appeared to be lighter than the background when the inducer surfaces were the palest figural units in the configuration. Polarity was reversed when this surface was the darkest. Therefore, the direction of the luminance gradient predicted the polarity of the illusion.

Both geometric and photometric factors thus seemed to play a role in creating the illusion. With acute angles, all observers reported the appearance of streaks in all luminance conditions. With 90° angles, illusory effects were recorded in four luminance combinations out of six. In addition, in these cases, judgments did not converge. With obtuse angles, the impression of streaks was very rarely evoked, and was totally absent for most observers. A first conclusion can thus be drawn: only corners having amplitudes of 90° or less are sources of illusory streaks. Data for inducers with obtuse angles were therefore excluded from further analyses.

**FIGURE 10:**
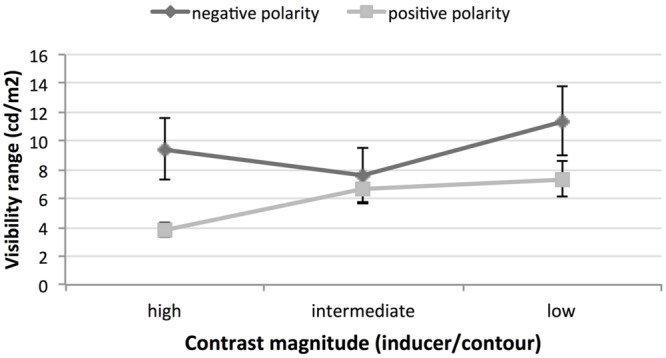
**Ranges of visibility of illusory streaks as function of contrast polarity and contrast magnitude.** Figures are averages across participants and direction of effect.

For each of the six conditions, **Tables 2** and **3** list the mean luminance coinciding with the appearance (T1) and disappearance (T2) of the illusion, and the difference between the two, which is the luminance range over which the effect persists. As mentioned previously, this range may be regarded as a measure of the vividness of the illusion. **Table 2** lists the results for right streak orientation and **Table 3** those for left (for plots of average ranges, collapsed across orientations, see **Figure 10**). As the two far right columns of both tables show, when observers perceived illusory streaks with inducers having right angles (i.e., square inducers), the mean values of the illusion visibility range tended to be lower than those recorded in acute angle conditions. In addition, with square inducers, the illusion was not perceived in the two conditions in which contour luminance was 24 cd/m^2^.

**Table 2:** Experimental results for three of four angular conditions, when orientation of streaks was from bottom-left to top-right.

Direction: *bottom-left to top-right*		Geometric conditions					
Photometric conditions		Acute angles I (46°)		Acute angles II (62°)		Square angles (90°)	
*Contrast polarity*	*Contrast magnitude class*	*Thresholds cd/m^2^*	*Range cd/m^2^*	*Thresholds cd/m^2^*	*Range cd/m^2^*	*Thresholds cd/m^2^*	*Range cd/m^2^*
Negative	High	25.8–34.6	8.8	26.8–37.3	10.5	–	–
	Intermediate	8.0–15.9	7.9	8.6–16.0	7.4	8.6–13.3	4.7
	Low	15–27.1	12.2	15.4–27.6	12.2	15.9–24.5	8.6
Positive	High	1.7–5.8	4.0	1.5–5.4	3.9	2.5–5.0	2.5
	Intermediate	6.5–12.8	6.3	5.7–12.1	6.4	7.9–10.8	2.9
	Low	17.2–24.4	7.1	15.3–23.5	8.2		

*For each of six combinations of contour-surface luminance (photometric conditions; see **Table 1** for detailed of luminance and contrast values), two results are given: (a) *thresholds*: upper and lower values of luminance (in cd/m^2^) of background in which illusory streaks persisted; (b) *range*: amplitude of luminance interval (difference between thresholds).*

**Table 3:** Experimental results for three of four angular conditions, when orientation of streaks was from top-left to bottom-right.

Direction: *top-left to bottom-right*		Geometric conditions					
Photometric conditions		Acute angle (46°)		Acute angle (62°)		Square angle (90°)	
*Contrast polarity*	*Contrast magnitude class*	*Thresholds cd/m^2^*	*Range cd/m^2^*	*Thresholds cd/m^2^*	*Range cd/m^2^*	*Thresholds cd/m^2^*	*Range cd/m^2^*
Negative	High	27.7–36.9	9.2	27.1–36.5	9.4	–	–
	Intermediate	7.9–14.8	6.9	8.7–16.9	8.2	8.6–11.7	3.1
	Low	15.2–25.6	10.5	15.3–25.8	10.5	15.9–23.4	7.4
Positive	High	1.8–5.6	3.9	1.9–5.3	3.3	2.9–4.5	1.6
	Intermediate	5.8–12.2	6.4	5.1–12.6	7.5	7.2–10.5	3.3
	Low	17.4–24.5	7.2	17.1–24.1	6.9		

*For each of six combinations of contour-surface luminance (photometric conditions; see **Table 1** for detailed of luminance and contrast values), two results are reported: *thresholds*: upper and lower luminance values (in cd/m^2^) of background in which illusory streaks persisted; *range*: amplitude of luminance interval (difference between thresholds).*

Although, these findings indicate the weaker persistence of illusory streaks in the 90° condition, the average range in this condition could not be formally compared with the average ranges recorded for the star-like inducers, because there was only partial agreement in observers’ estimates. Also, a complete set of judgments could only be gathered when the inducers had acute corners. Consequently, only data for acute angle conditions were further analyzed.

#### Acute Angles (Star Inducers)

Data on the range of visibility of the effect were analyzed in a four-way, repeated-measures ANOVA including *angle amplitude* (46 and 62°), *contrast polarity* (positive or negative), *magnitude of the Michelson contrast* between inducer surfaces and contours (three levels; see **Table 1**) and *direction of illusory streaks* (left = top-left to bottom-right; right = bottom-left to top-right). The results showed two significant main effects (*polarity* and *magnitude*) and a significant two-way interaction between these factors. No significant differences were found in the vividness of the illusion across angle amplitudes or directions. No further significant interaction among any other factors in the ANOVA was found.

**Figure 10** shows visibility ranges as functions of contrast polarity and contrast magnitude, averaged across participants and the direction of the effect. The extent of the range when streaks persisted was significantly greater [*F*(1.19) = 22.38; *p* < 0.001] for the dark polarity (9.5 cd/m^2^) than for the pale one (5.9 cd/m^2^). The range of illusion persistence was then found to increase significantly [*F*(2.38) = 17.26; *p* < 0.001] as the Michelson *contrast between the star surface and its contour* decreased (average ranges for high, mid, and low contrast were, respectively, 6.6, 7.1, 9.3 cd/m^2^).

The interaction between streak polarity and contrast magnitude was also significant [*F*(2.38) = 17.21; *p* < 0.001]. Although, the range of visibility of the light streaks was linearly related to the intensity of the luminance contrast, this did not occur for the dark streaks.

It is important to note that, in 36.8% of the trials, phantom streaks were seen even when the contour was isoluminant with the background (i.e., one of the two thresholds coincided with a starting or ending point of the series). This means that the luminance gradient is not a necessary condition for the creation of the illusion. Interestingly, participants reported this effect in the presence of a luminance step (the edges separating the inside of the inducers and the square, given the isoluminance of square and contour) only when the contrast at this edge was low (**Table 4**).

**Table 4:** Frequency of trials in which effect was perceived, even when contour was isoluminant with square in which streak was seen.

	Negative contrast polarity			Positive contrast polarity		
Angle amplitude (°)	High contrast	Intermediate contrast	Low contrast	High contrast	Intermediate contrast	Low contrast
46	5	25	33	15	27	21
62	0	16	29	10	23	12
*% participants*	0,0625	0,5125	0,775	0,3125	0,625	0,4125

*First two rows show number of participants in each condition who reported perceiving the effect. Last row, for combination of contrast polarity and contrast magnitude, shows numbers of participants reporting effect, even when contour luminance was equal to square luminance.*

## Discussion

The experiment reported here was conducted to examine a novel grouping effect occurring in a repetitive pattern, which takes the form of illusory bands connecting the corners of oriented inducers. Our test results confirm that the luminance contrast at the corners and their amplitude are relevant to the appearance of streaking lines and affect the luminance range of visibility of the illusion. Our main findings may be summarized as follows:

(i) Illusory streaks are seen to join angles of 90° or less. The phenomenon is always recorded when the angles are acute, although when the stimulus contour is a square, the illusion occurs in most cases but with discordant judgments on the part of subjects. Wider angles eliminate this effect.
(ii) The range of visibility of the illusion is not linearly related to angle amplitude. However, the weakest manifestations were recorded with square inducers.
(iii) The streaks have the same contrast polarity as the corner surfaces.
(iv) A luminance ramp at corners strengthens the illusion, as the highest intensities are attained when smooth gradients (surface-contour-surround) are shown. However, even in the case of isoluminance between contour and surround, illusions were perceived.
(v) The darker streaks persist over a larger range of variations of the background.

**FIGURE 11:**
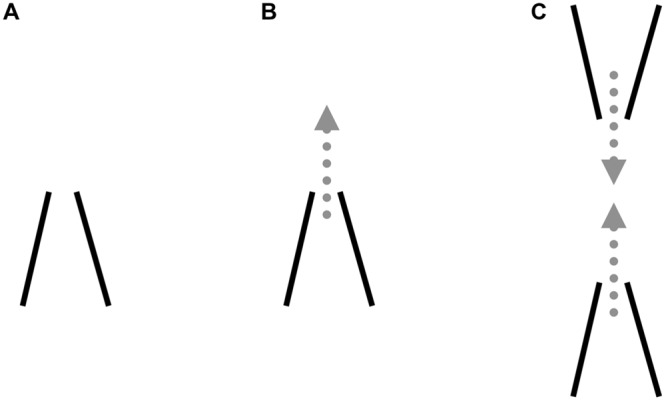
**Local and global factors producing illusory streaks.** Local: **(A)** Boundary gap at corner vertex when two conditions are met, i.e., low contrast and angle sharpness; **(B)** Color spreading occurs within definite borders and flows into surround when a border gap forms. Global: **(C)** Converging spreads from opposite corners. Arrows: merging of two concomitant diffusion phenomena.

First, these findings help to clarify the geometric and photometric conditions in which illusory streaks arise, although uncertainty still persists regarding their causes and origin. Why does a sharp, low-contrast corner behave like a geyser spouting featural qualities of the shape it defines? If color spreading occurs, why does it concentrate in the vertexes but is absent in the straight edges of the inducers? We believe this phenomenon may be explained by examining both local and global processes, as shown in **Figure 11**.

### Local Effects

Two main phenomena seem to be locally at play at the inducer corners:

(a) A boundary gap at the corner vertex (**Figure 11A**). When two conditions are met, i.e., low contrast and angle sharpness, we assume that contour processing has been interrupted or is malfunctioning. We cannot provide empirical support to this assumption, since we could not find any research demonstrating anything similar. The end-stopping units theorized by Grossberg and Mingolla (1985a,b) in such geometrical and photometric conditions seem to prevent the representation of a closed contour in the boundary contour system.
(b) Color spreading. The dotted arrow in **Figure 11B** indicates diffusion of inner color from the boundary gap at the vertex. In accordance with the model of Grossberg and Mingolla (1985a,b), we assume that surface filling occurs within definite borders and flows into the surround when a border gap forms.

### Global Effects

The far right figure (**Figure 11C**) shows that convergence spreads from opposite corners. The two arrows symbolize the merging of the diffusion phenomena giving rise to illusory streaks.

We now examine the implications of this theory as related to the processes of *contour formation* and *perceptual grouping*, in the light of our findings and a literature review on related illusions.

### Contour Formation

Grossberg and Mingolla (1985a,b) provide a detailed description of the “end-cutting process” at the end-points of a line or the ends of a bar. These processes are required in the region of a border where contour signals are weak or absent, to prevent surface feature signals from flowing into the surround. The “end-cutting process” acts as a bank, preventing surface filling. The Authors do not explicitly describe the effect of these processes at corners instead of line endings, but they believe that the same processes occur both at line endings and at corner regions. In the latter case, processing at the angle location would be required, to “embank” color propagation outwardly from the vertex.

The authors assume that “In order to work properly, boundary contour responses need to be sensitive to the amount of contrast in scenic edges” (Grossberg and Mingolla, 1985a, p. 182). Our hypothesis is that, at low contrast, end-cutting processes do not reach a good result: the vertexes of the acute angles are not properly perceived and a gap forms at the inducer boundaries. The streaks in **Figures 1A** and **3B** can thus be viewed as the results of filling-in signals, spreading the features of the inner surface of the inducers round the corners, along their bisectors. This assumption correctly predicts the polarity and location of the streaks reported in the experiment and in phenomenological observations.

To our knowledge, the illusory alteration in brightness in the space outside the vertex has not been previously recorded. Brightness alterations at corners were previously documented by Troncoso et al. (2005, 2006, 2009) but only *in the inside surface* (**Figure 6D**) and their magnitude was inversely related to angle width. An exception may be the illusory spot at the convergence of the square corners in the Hermann grid (**Figure 6C**) and in the pincushion illusion of Schachar (1976) but, again, the conditions and effects are different from the streaking illusion illustrated here (see Spillmann, 1994, for a review). Hermann spots and Schachar diagonals are in fact generated in a context of high contrast (Morgan and Hotopf, 1989), are documented with right angles, and have different phenomenological features. To mention only one, they do not appear as a continuous segment connecting two vertexes.

Illusory phenomena of color induction and/or bright ness/color assimilation have frequently been observed in the photometric conditions which appear to be crucial for illusory streaks to appear, i.e., low luminance contrast between an outline contour, or *fringe*, and the background (Broerse et al., 1999). These brightness/color induction effects are generated by lines or dots (Kitaoka, 2001; Pinna and Grossberg, 2005), and thus similar phenomena may also occur all round the contour of an angle. However, if the same fringe-induced mechanisms were involved in our illusion, we would not expect the effect to be enhanced at the vertex, as it is with streaks. In addition, one particular finding mentioned above challenges a fringe-induced interpretation: the streaks also propagate from luminance discontinuities, not only from luminance ramps. In other words, they are seen even in conditions in which fringe-induced phenomena do not arise.

Therefore, according to the reviewed literature, we cannot predict the appearance of phantom streaking lines connecting the inducer corners in **Figures 1A** and **3B** and experimental stimuli. Our hypothesis is that boundary formation is prevented when a sharp corner must be perceptually completed. These difficulties are greater when corners are narrower than 90°, perhaps because, the sharper the corners, the more similar they are to line ends, which are the features which should trigger activity in end-stopping cells. In view of our results, we could also speculate whether interactions between end-stopping cells and boundary ownership signals from the BC System play a role in the illusion. Overall, however, these processes and the conditions in which they operate are still relatively unexplored, and further research is required to increase our understanding.

### Grouping

In our view, illusory diagonals are the result of a global effect arising from local phenomena. Two suggestions may be made.

The diagonal strikes documented here are probably a manifestation of the ease with which regions of brightness alteration merge to give rise to illusory “streets” in visual space. Several of the related illusory phenomena which we have illustrated, such as the Van Tujil, Ehrenstein, and Vasarely Arcturus configurations (**Figures 6A–D**), can indeed be explained in terms of “summation” of local brightness alterations originating in the locations of the figural units periodically repeated across the visual field. As noted by Grossberg and Mingolla (1985a,b), complete, stable figural organization may coexist with organization processing which gives rise to illusory effects such as phantom contours. Paradoxically, a strong organization like a regular repetitive pattern may actually reinforce concurrent organizations by multiplying weak local flaws or misperceptions.

However, mere summation seems insufficient to account for our illusion. Illusory streaks are in fact not only not seen in a corner in isolation (**Figure 5**), but their appearance also seems to be related to the alignment of corner axes and the spatial separation of vertexes. These three factors – alignment, distance, and number of local units – lead us to hypothesize that, at the vertexes of the star-like shapes in **Figures 7–9**, something similar to lines or oriented bands is perceived. A rich literature exists on the roles of alignment, proximity and number in the perceptual grouping of line fragments, and experiments on “contour detection” processes have generated a considerable amount of data. The perceptual task in these experiments generally consists of identifying the presence of a chain of Gabor patterns aligned along a path within a set of randomly arranged similar configurations (Field et al., 2000). We may assume that the same perceptual links that allow the Gabors to emerge as whole units are active even in the checkerboard patterns of our experimental stimuli. Hess and Dakin (1999, p. 956) also observed that, in peripheral vision, Gabor units arranged to form a straight path “appear as blurred luminance defined lines” and a phenomenological feature assimilates these illusory effects to those we document here. However, the above authors attributed this perceptual outcome to linear filtering processes, rather than to grouping operations acting on Gabor units nearby.

The illusory effects explored here may also be related to another grouping factor, reported by Palmer and Rock (1994) and called *connectedness*. According to this principle, for example, pairs of dots connected by lines are organized as subgroups, even in violation of Gestalt proximity and similarity grouping laws. We have demonstrated that two corners close to each other do not generate a visible illusory link, but several of them do. The hypothesis is that a chain of local illusory effects makes the streaks visible, and that they in turn reinforce the grouping between the inducers they connect.

Further research is certainly required to test the role of Gestalt grouping factors (e.g., “good continuation”) and other unexplored merging factors, in our illusion and in other similar effects involving phantom “streets” or lines, such as the Ehrenstein, Van Tujil, and Kitaoka configurations. Two sets of factors, as we have seen, are probably involved in the appearance of phantom diagonals, but both their relative effect and their precise role in the emergence of the streaks is still unclear. We are currently designing and planning some experiments aimed at investigating further the role of good continuation and proximity in this illusory phenomenon, which may shed more light on it and its determinants in the visual system. Further insights may come from studies of end-stopping processes. In any event, we believe that this and other illusory effects should be analyzed in depth before we can reach full understanding of perceptual grouping, as they may reveal still unexplored aspects of visual organization, eventually leading to revisions or extensions of current models.

## Author Contributions

SR: contributed to designing the experiment, analyzing the data, and writing the paper; SG: contributed to writing the paper and analyzing the data; OP: contributed to writing the paper; LB: contributed to conducting the experiment.

## Conflict of Interest Statement

The authors declare that the research was conducted in the absence of any commercial or financial relationships that could be construed as a potential conflict of interest.
