# Supplementary material for: Illusory Streaks from Corners and Their Perceptual Integration
Source: Front Psychol. 2016 Jun 23;7:959. doi: 10.3389/fpsyg.2016.00959 (PMC4917560; doi:10.3389/fpsyg.2016.00959)

Appendix

A synthesis if the procedure of stimuli production. A portion of the whole stimulus configuration is depicted made up of four checks (or quadrants) and a superimposed inducer. The columns illustrate the results of manipulations of the luminance of a basic figure (top figures).

Top figures: the contour and two opposite quadrants (indicated by stars and cross symbols) are isoluminant

Going downwards: the isoluminant quadrants change in luminance so that to form a luminance ramp. If the square is darker than the contour then the quadrants (indicated by white stars) will progressively lighten (left column). If the square is lighter than the contour then the quadrants (indicated by black crosses) will progressively darken (right column). The outline stairs symbolize the luminance profile at the border of squares (dotted horizontal line). Note that in the quadrant invariant in luminance the contour is the darkest or the lightest figural element.

The five configurations illustrate a selection of the stimuli there were created by progressively changing the grey shade of the quadrants.

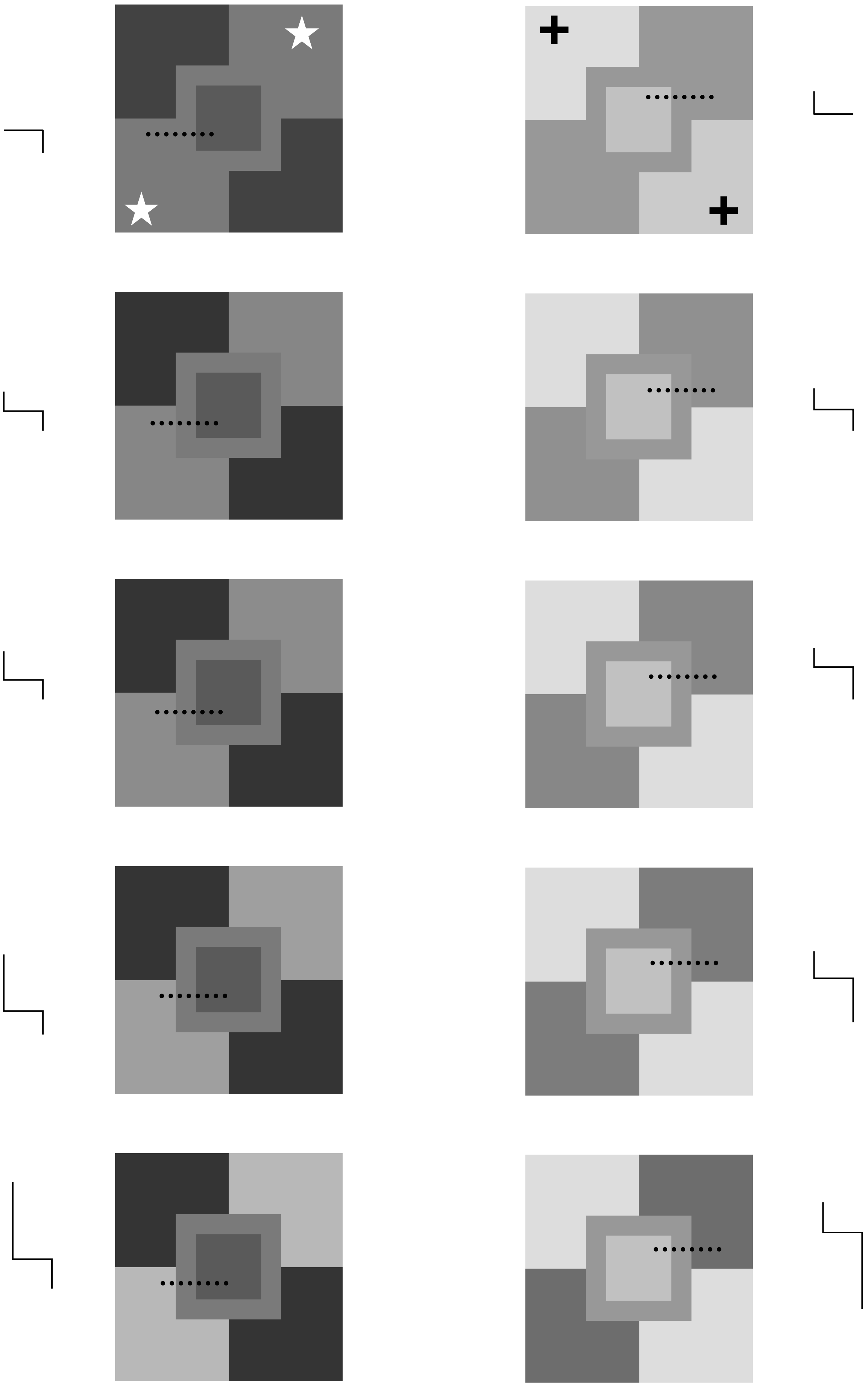

Supplement: Supplementary file 1 [file Image_1.PDF]
